# *Staphylococcus aureus* Augments Epithelial Skin Barrier Damage Through T Cell Activation in Cutaneous T Cell Lymphoma

**DOI:** 10.1111/all.70292

**Published:** 2026-03-08

**Authors:** Maria Gluud, Emil M. Pallesen, Ziao Zeng, Martin R. J. Namini, Chella Krishna Vadivel, Lise Mette Rahbek Gjerdrum, Lang Yan, Sana Ahmad, Lise M. Lindahl, Michael Bzorek, Maria R. Kamstrup, Saptaswa Dey, Sisse Rye Ostrowski, Erik Sørensen, Pia Rantakari, Charlotte Menne Bonefeld, Carsten Geisler, Anders Woetmann, Riitta Lahesmaa, Lars Iversen, Peter Wolf, Thomas Litman, Terkild B. Buus, Niels Ødum

**Affiliations:** ^1^ Department of Immunology and Microbiology, LEO Foundation Skin Immunology Research Center University of Copenhagen Copenhagen Denmark; ^2^ Department of Pathology Zealand University Hospital Roskilde Denmark; ^3^ Department of Clinical Medicine, Faculty of Health and Medical Sciences University of Copenhagen Copenhagen Denmark; ^4^ Department of Dermatology Aarhus University Hospital Aarhus Denmark; ^5^ Department of Dermatology Bispebjerg and Frederiksberg Hospital Copenhagen Denmark; ^6^ Department of Dermatology and Venereology Medical University of Graz Graz Austria; ^7^ Department of Clinical Immunology Copenhagen University Hospital, Rigshospitalet Copenhagen Denmark; ^8^ Turku Bioscience Centre, University of Turku and Åbo Akademi University Turku Finland; ^9^ InFLAMES Research Flagship Center University of Turku Turku Finland; ^10^ Institute of Biomedicine, University of Turku Turku Finland; ^11^ BioTechMed Graz Graz Austria

**Keywords:** barrier, epithelium, infections, inflammation, T cells

## Abstract

**Background:**

Skin barrier dysfunction is central to inflammation and susceptibility to infection in atopic dermatitis (AD). Cutaneous T‐cell lymphoma (CTCL) shares clinical similarities with AD and is also associated with a high prevalence of *Staphylococcus aureus* ( *S. aureus* ) colonisation. However, the mechanisms driving skin barrier damage in CTCL and the contribution of bacteria remain poorly understood.

**Methods:**

We investigate how the interplay between *S. aureus* (and staphylococcal enterotoxins (SEs)) and primary malignant‐ and non‐malignant T cells affects keratinocyte expression of skin barrier proteins; in vitro, in an EL4 murine lymphoma model of bacteria‐driven tumour progression, and in CTCL patient lesions colonised with SE‐producing *S. aureus* before and after bacterial eradication by antibiotic treatment.

**Results:**

*S. aureus* and SEs activate malignant and non‐malignant T cells to release barrier‐repressing cytokines, including IL‐4, IL‐13, IL‐22, and OSM, and JAK‐dependent downregulation of filaggrin and loricrin in keratinocytes. In the EL4 model, bacteria‐colonised tumour‐bearing mice show significant filaggrin loss in tumour‐adjacent epidermis, whereas antibiotic‐treated mice maintain near‐normal expression. Clinically, antibiotic eradication of SE‐producing *S. aureus* partially restores filaggrin and loricrin expression in three of four patients, paralleling reduced inflammatory signalling.

**Conclusions:**

SE‐producing *S. aureus* promotes skin barrier impairment in CTCL through cytokine‐driven, JAK‐dependent repression of structural proteins in keratinocytes. These findings identify microbial–immune crosstalk as a contributor to CTCL skin pathology and provide mechanistic rationale for strategies targeting *S. aureus* colonisation as adjunctive therapy in CTCL.

## Graphical abstract

*Staphylococcus aureus,* commonly colonising CTCL patients, augments skin barrier dysfunction. Staphylococcal enterotoxins induce T‐cell release of barrier‐repressing cytokines (IL‐4, IL‐13, IL‐22, OSM). Cytokine signalling drives JAK‐dependent downregulation of filaggrin and loricrin in keratinocytes. Antibiotic‐mediated eradication of *S. aureus* induces skin barrier protein expression and inhibits the inflammatory cycle. CTCL, cutaneous T cell lymphoma; FLG, filaggrin; LOR, loricrin; OSM, oncostatin M; *S. aureus* , *Staphylococcus aureus* ; SE, staphylococcal enterotoxin.

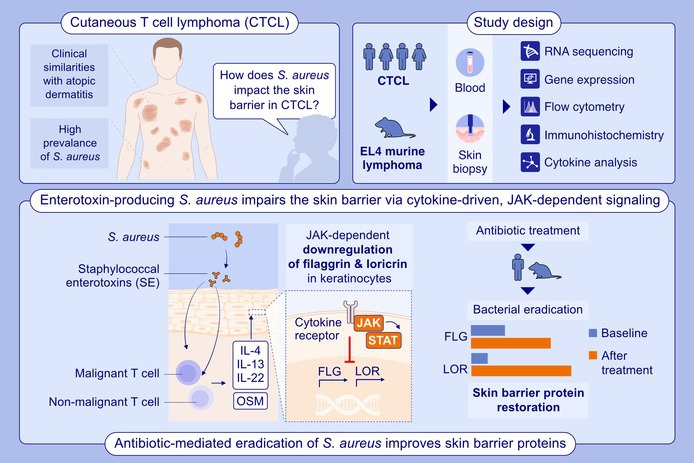

## 1 Introduction

A healthy epithelial barrier is essential in protecting the host from external insults and disease [1, 2, 3]. The pathogenic role of a compromised skin barrier is well established in allergic diseases such as asthma and atopic dermatitis (AD) [1, 4, 5, 6, 7]. Filaggrin proteins (filaggrin and filaggrin‐2) play important roles in maintaining skin barrier integrity and function [5, 8, 9, 10, 11, 12]. Loss of function mutations of filaggrin is a major risk factor for AD and inflammation‐driven defects of filaggrins are associated with a compromised skin barrier function including increased susceptibility to *Staphylococcus aureus* ( *S. aureus* ) infections [8, 13, 14, 15, 16, 17].

Cutaneous T‐cell lymphoma (CTCL), including Mycosis Fungoides (MF) and Sézary syndrome (SS), is characterised by malignant T cells residing in chronically inflamed skin lesions and also the presence of malignant cells in the blood for SS [18, 19, 20, 21, 22, 23]. CTCL shares clinical and molecular features with benign inflammatory diseases, in particular with AD [24, 25]. CTCL lesions have a deficient skin barrier as judged by reduced filaggrin expression and enhanced trans‐epidermal water loss compared to non‐lesional skin sites [26, 27, 28]. Epidermotrophic malignant T cells orchestrate a TH2‐dominated inflammatory environment that induce JAK/STAT3‐driven changes in keratinocytes suppressing filaggrin expression [26, 29, 30, 31].

CTCL lesions are frequently colonised by *S. aureus* , and CTCL patients have an elevated risk of sepsis and pneumonia, particularly after diagnosis [32, 33, 34, 35, 36, 37, 38, 39, 40, 41, 42, 43, 44, 45]. *S. aureus* colonisation has been consistently linked to increased disease activity [35, 46, 47, 48, 49, 50, 51, 52]. Yet the mechanisms by which *S. aureus* affects CTCL skin lesions and compromises the skin barrier remain poorly understood.

In the present study, we show that patient‐derived and purified Staphylococcal enterotoxins (SEs) induce cytokine production in CTCL cells that suppresses key skin‐barrier proteins. Conversely, antibiotic‐mediated eradication of *S. aureus* reduced cytokine expression and restored barrier proteins in three of four patients. These findings support a vicious cycle in which *S. aureus* colonisation activates malignant T cells, drives barrier damage, and promotes further bacterial overgrowth. This mechanism may also be relevant to other inflammatory skin disorders characterised by barrier dysfunction and increased susceptibility to *S. aureus* infection.

## 2 Materials and Methods

### 2.1 *Staphylococcus aureus* Cultures

As described [53] *S. aureus* cultures isolated from SS (SA.1, SA.3) and MF (SA.2) patients were grown in TSB medium in a density OD_600nm_ = 0.01 for 4 h with or without antibiotics (meropenem, 8 μg/mL) or 1 μg/mL of the *S. aureus* targeting compounds Endolysin XZ.700, MEndoB, MEndoB‐mutant (Micreos Group, The Netherlands).

### 2.2 Patient Material

Samples from two cohorts of patients were applied in the study. Paired non‐lesional and lesional skin biopsies from four patients diagnosed with advanced stage MF (MF1‐MF4, IIB‐IIIA) and undergoing treatment for 24 days with antibiotics; 10 days with intravenous antibiotics (cephalosporin and metronidazole) followed by oral treatment for 14 days with a combination of amoxicillin and clavulanate at Aarhus University Hospital [35]. Patients were *S. aureus* positive (none had MRSA) and further characteristics and concomitant therapies are listed in Table S1. Biopsies were from the same target skin lesion before initiation (Day 0) of antibiotic treatment and after 2 months of antibiotic treatment (Day 60) [35]. Blood samples from patients diagnosed with SS (SS1‐SS6) and one patient diagnosed with leukaemic MF were provided by Bispebjerg Hospital. All experiments were performed after informed consent from the patients and in accordance with the Declaration of Helsinki. Samples were obtained after approval by the Regional Ethical Committee, Denmark (1‐10‐72‐151‐16/M‐20090102/H‐16025331).

### 2.3 Peripheral Blood Mononuclear Cell (PBMC) Culturing and Flow Cytometry Analysis

PBMCs from patients with SS or MF (CTCL cells) were isolated by density gradient centrifugation and cultured in RPMI‐1640 supplemented with 10% human serum and 1% Penicillin/Streptomycin (PBMC media) for 4 h‐144 h with PBS, a pool of SEs (SE‐pool); 50 ng/mL of each of; SEA/SEB/SEC1/SED/SEE or individual SEs (Toxin Technology, concentrations within physiological range [54, 55]). CTCL cells were cultured for 1.5 h in 3 parts PBMC media to one part bacterial medium TSB, *S. aureus* supernatant, or supernatants derived from *S. aureus* treated with antibiotics or Endolysin, followed by 72 h incubation. Flow cytometry analysis on the BD‐LSR‐Fortessa flow cytometry was performed and malignant T cells were gated as TCRvβ18^+^CD26^−^CD3^+^CD4^+^.

### 2.4 Normal Human Epidermal Keratinocytes (NHEK)

Several vials of NHEK (adult, pooled donors) were cultured in Basal keratinocyte medium containing growth factors (Promocell, C20111), and 1.25 mM CaCl_2_ when differentiating prior to experiments.

### 2.5 ELA‐4 Murine Model

Six C57BL/6 mice were inoculated intradermally with EL4 cells and three were treated with antibiotics (Neosporin (neomycin/bacitracin/polymyxin B sulphate)) and three mice treated with vehicle (Vaseline petroleum jelly) as previously described [51]. Two healthy mice (without EL4) were included, one of which was treated with Neosporin and one with vehicle (Vaseline). Additional information and mouse characteristics are included in Table S2 [51].

### 2.6 Quantitative Reverse Transcription PCR (RT‐qPCR)

RNA purification and RT‐qPCR analysis were performed as previously described [26]. β‐actin was used as reference genes for normalisation and data was calculated according to the $2^{∆∆\mathrm{Ct}}$ method.

### 2.7 Cytokine Analysis

Mesoscale (MSD) V‐plex assay kits and Duoset ELISA (R&D) were used to analyse cytokines in supernatants from CTCL cells.

### 2.8 Cellular Indexing of Transcriptomes and Epitopes by Sequencing (CITE‐Seq)

Previously published CITE‐seq. data sets of PBMCs cultured in the presence of PBS or SE‐pool for 48 h were reanalysed for cytokine expression [56, 57] as described in Supporting Information and elsewhere [58, 59, 60]. Malignant T cells were identified based on their monoclonal T‐cell receptor (TCR)‐β CDR3 region (from TCRαβ modality) and high expression of malignant‐associated genes [61].

### 2.9 Immunohistochemistry (IHC)

For IHC studies pY‐STAT3 (Tyr705, 1:800, D3A7, Cell Signalling, #9145L), pY‐STAT1 (Tyr701, 58D6, Cell signalling), pY‐STAT6 (pY641, 18/P‐STAT6, BD bioscience), filaggrin (GeneTex, #GTX23137, 1:50), filaggrin‐2 (Aviva Biosystems, #OACD03572, 1:4000) were used. The procedure is described in Supporting Information and elsewhere [35].

### 2.10 Imaging Mass Cytometry (Hyperion)

Deparaffinised sections were treated at pH‐6, stained with the primary filaggrin antibody, a secondary metal‐conjugated antibody, and subsequently an antibody mix comprising all the metal‐conjugated antibodies. Regions of interest (1.0 mm^2^) were analysed by Hyperion imaging system. The MCD viewer was applied to study the images. Table S3 lists antibodies included.

### 2.11 Ingenuity Pathway Analysis (IPA) of Cytokine Signalling

Transcriptomic profiling of CTCL patient biopsies was performed using the Affymetrix GeneChip Human Transcriptome Array 2.0 (Rigshospitalet, Denmark) as previously described [35]. Cytokine pathway activity was assessed using gene‐enrichment–based analyses in Ingenuity Pathway Analysis (IPA, Qiagen). Upstream Regulator Analysis was applied, and cytokine network activity was inferred from the IPA activation *z*‐score, with *z* > 2 indicating predicted activation and *z* < −2 indicating predicted inhibition.

### 2.12 Statistics

All graphs and statistical analysis were performed using the GraphPad‐Prism v8.0 software. Student's *t*‐test and one‐way analysis of variance were applied, followed by post hoc analysis. For qPCR results the relative expression of each intervention was compared to the control. Error bars are shown as SEM, and the level of statistical significance was set to *p*‐value < 0.05 (*).

## 3 Results

### 3.1 *S. aureus* ‐ and SE‐ Stimulated CTCL Cells Repress Filaggrin Expression in Keratinocytes In Vitro

**FIGURE 1:**
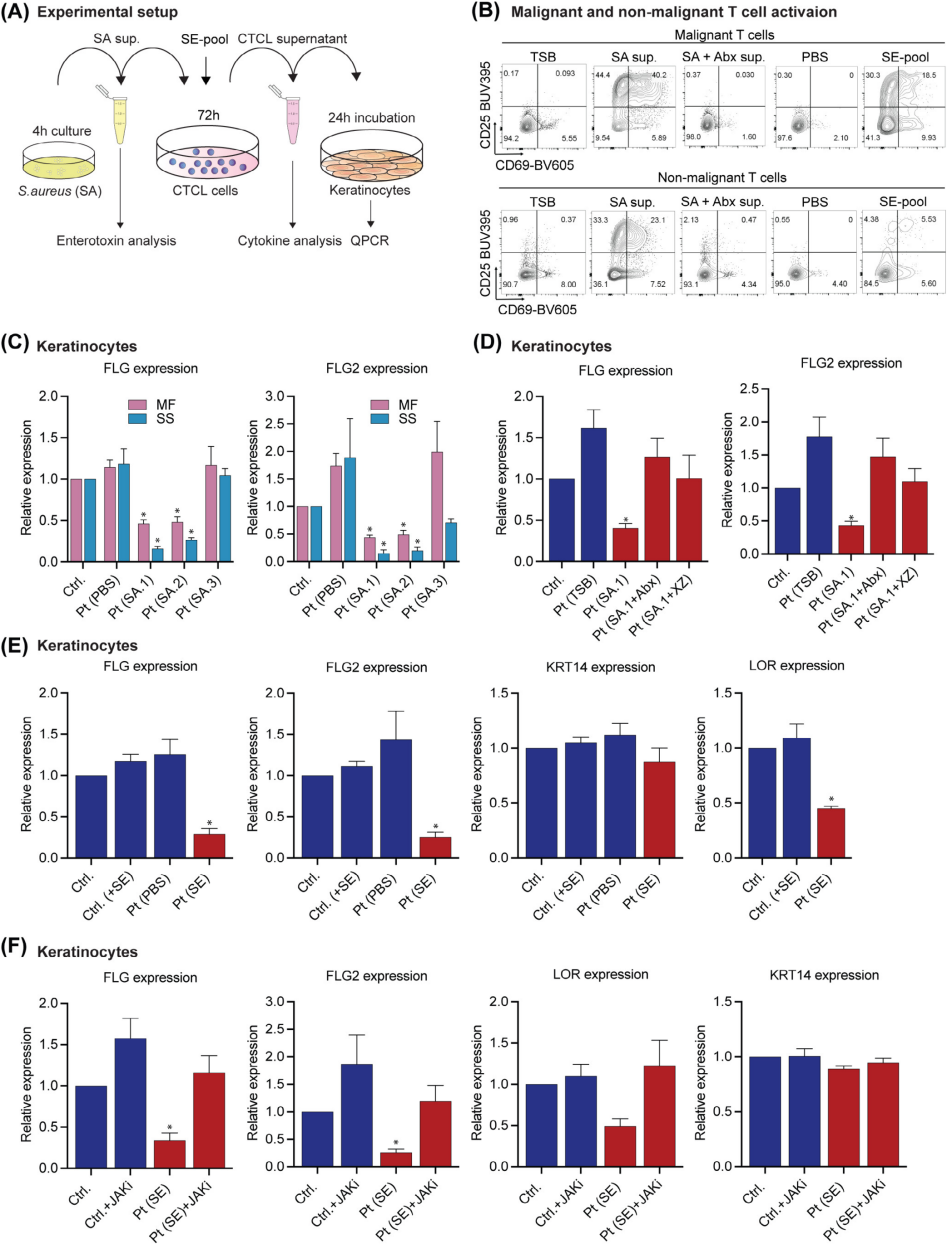
*S. aureus* ‐ and SE‐stimulated CTCL cells repress filaggrin expression in keratinocytes. (A) An illustration of the experimental setup is presented. To stimulate CTCL patient PBMC's (CTCL cells) isolates from SE‐producing *S. aureus* were prepared and transferred to the cells for 1.5 h, followed by washing and further culturing for 72 h or a pool of purified SEs was transferred to the CTCL cells for 72 h. (B) Flow cytometry analysis demonstrating activation (CD25^+^ and CD69^+^ expression) of CTCL cells following stimulation. Malignant T cells were determined based on TCRvβ18^+^CD26^−^CD3^+^CD4^+^ for the one patient presented. (C) mRNA expression analysis of NHEK stimulated with CTCL media (Ctrl.), or supernatants from CTCL cells (either from one MF or one SS patient) cultured in PBS (Pt(PBS)) or bacterial isolate SA.1 (Pt‐SA.1), SA.2 (Pt‐SA.2), or SA.3 (Pt‐SA.3). Data shows three independent experiments (*n* = 3). (D) mRNA expression analysis of NHEK stimulated with CTCL media (Ctrl. media), supernatants from CTCL cells (three different patients) cultured with bacterial TSB media (Pt(TSB)), supernatant from patient‐derived *S. aureus* (Pt(SA)), *S. aureus* pre‐treated with antibiotics (Pt(SA + Abx)) or *S. aureus* pre‐treated with endolysin, XZ700, (Pt(SA + XZ)). (E) mRNA expression analysis of NHEK stimulated with supernatants from CTCL cells cultured with a pool of purified SEs (Pt(SE)) (five patients). (F) mRNA expression analysis of NHEK cultured with supernatants from SE‐stimulated CTCL cells (five patients) in the presence or absence of 10 μM JAK inhibitor or DMSO control. (C–E) mRNA expression levels of FLG, FLG2, LOR and KRT14 using β‐Actin as a reference gene were given as relative expression. (*) indicates statistical significance (*p* < 0.05).

Cytokines have recently been linked to decreased expression of filaggrin in CTCL [26]. Since SEs are potent T‐cell activators [54] we hypothesised that SE‐induced cytokines from CTCL cells could repress barrier‐related proteins filaggrin (FLG), filaggrin‐2 (FLG2), and loricrin (LOR) mRNA expression in keratinocytes (NHEK) [5, 48, 62]. The experimental setup is illustrated in Figure 1A. Supernatants from PBMC's obtained from CTCL patients (CTCL cells) stimulated with SE‐producing *S. aureus* isolates obtained from lesional CTCL skin from SS (SA.1) and MF (SA.2) were tested alongside with supernatants from CTCL cells cultured in the presence of a pool of purified SEs (SE‐pool) containing the SEs; SEA, SEB, SEC1, SED and SEE (Figure 1A–F). Flow cytometric analysis of CTCL cells treated with either *S. aureus* supernatants, or the SE‐pool, demonstrated activation of both malignant and non‐malignant T cells by flow cytometry analysis as judged by CD25 and CD69 upregulation, which was abrogated by antibiotics (Figure 1B; Figure S1A). NHEK treated with supernatants from *S. aureus‐*stimulated CTCL cells (both MF and SS) displayed a profound decrease in the expression of FLG and FLG2 when compared to NHEK treated with supernatant from control treated cells and untreated (media‐treated) NHEK (Figure 1C). The two *S. aureus* isolates (SA.1 and SA.2) containing SEA and also SEE for SA.1 (confirmed by ELISA) had similar effects whereas the non‐SE producing *S. aureus* isolate (SA.3) did not affect the expression (Figure 1C). Addition of antibiotics or specific anti‐ *S. aureus* bacteriophage‐derived endolysins (XZ.700 and the enhanced chimeric‐endolysin MEndoB [63]) blocked the subsequent repression of FLG and FLG2 (Figure 1D; Figure S1C). The effect was selective as keratin‐14 (KRT14) expression by keratinocytes was largely unaffected (Figure S1B). In support, stimulation with an SE‐pool fully replicated the effect of supernatant from live *S. aureus*. Thus, supernatant from SE‐stimulated CTCL cells repressed expression of FLG, FLG2 and LOR in NHEK, without affecting KRT14 (Figure 1E). Moreover, both SEA and SEE were observed to have an effect individually (Figure S1D–F). The observations were confirmed on the protein level where expression of filaggrin and filaggrin‐2 were downregulated in a reconstructed epidermal model (Figure S1G).

### 3.2 JAK Inhibition Largely Abrogated SE‐Induced Repression of Filaggrins in Keratinocytes

We then assessed the effect of the pan‐JAK inhibitor, Tofacitinib, by adding supernatants from SE‐stimulated CTCL cells to NHEK, in the presence or absence of JAKi. As shown above, supernatants from SE‐treated CTCL cells significantly suppressed keratinocyte expression of FLG, FLG2, and LOR (Figure 1F). This repression was largely blocked by Tofacitinib, suggesting that the downregulation was primarily mediated by cytokine‐induced JAK/STAT activation.

### 3.3 SEs Induce Expression of Filaggrin‐Repressing Cytokines in CTCL Cells

**FIGURE 2:**
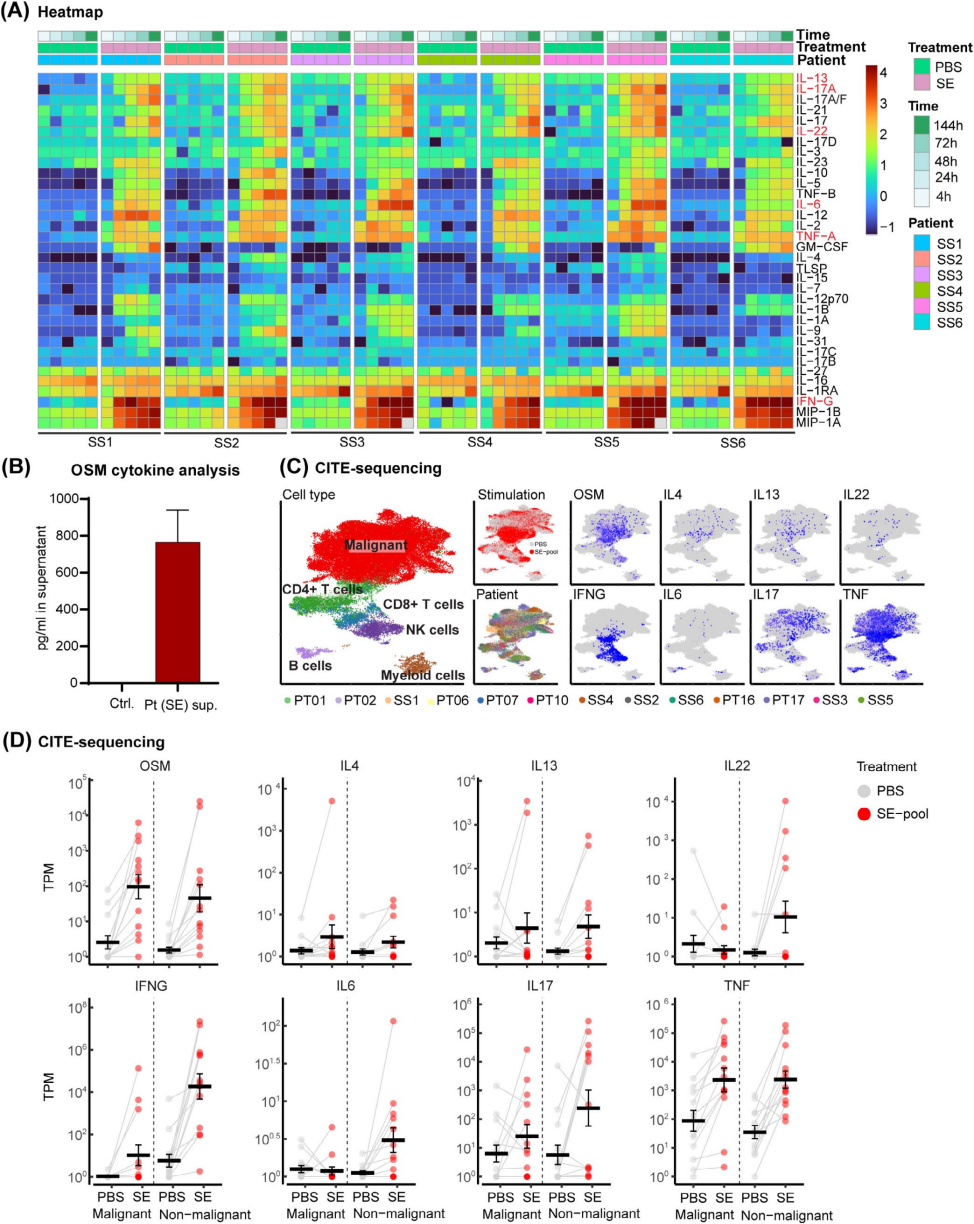
SEs induce expression of filaggrin‐repressing cytokines in CTCL cells. (A) Mesoscale analysis to detect the presence of cytokines in the supernatants collected from PBMCs obtained from six SS patients (CTCL cells, SS1‐SS6) stimulated with PBS or a SE‐pool for 4, 24, 48, 72, 144 h. The values illustrated in the heatmap were given as log_10_ pg/mL. (B) OSM protein (pg/mL) ELISA of supernatants from three of the CTCL patients stimulated with a SE‐pool for 72 h. (C) UMAPs illustrating re‐analysis of single‐cell RNA‐sequencing data for the six CTCL patients (SS1‐SS6) as well as seven additional patients stimulated with the SE‐pool for 48 h. (D) Cite‐seq. data of cytokine expression within malignant and non‐malignant cell types was visualised as transcripts per million (TPM).

To elucidate whether SE‐activated CTCL cells produce cytokines known to repress filaggrins in NHEK, we measured cytokines in supernatants from six leukaemic SS patients (SS1‐SS6) (CTCL cells) stimulated with PBS or the SE‐pool. IL‐6, IL‐13, IL‐17, IL‐22, IFN‐γ, and TNF‐α accumulated over time in supernatants from SE‐treated cultures when compared to controls (Figure 2A). Similar cytokines were observed when stimulating CTCL cells with the bacterial isolate (SA.1) and when stimulating PBMC's from a leukaemic MF patient with the SEpool (Figure S2A,B). It was not unexpected [26, 64, 65] that the kinetics and relative expression of these cytokines differed between patients, but overall, the cytokines in question increased over time in all patients tested. An OSM‐specific ELISA detected high levels of OSM in CTCL cells stimulated with SE‐pool for 72 h (Figure 2B). To address which cell types expressed individual cytokines, we analysed public CITE‐sequencing data of the above mentioned six patients and seven additional patients—all from previously published studies (Figure 2C,D) [56, 57]. Both malignant/non‐malignant T cells were induced to express TH2‐associated cytokines (IL‐13 and OSM) and TNF‐α whereas IL‐6 and IL‐22 were almost exclusively seen in non‐malignant cells. IFN‐γ and IL‐17 were induced in both malignant and non‐malignant cells, but generally stronger in the latter (Figure 2C,D). These findings indicate that SEs induce secretion of a series of cytokines, many of which are linked to JAK/STAT‐mediated repression of filaggrins in keratinocytes [26, 66].

### 3.4 Improvement of Filaggrin and Loricrin Expression in Skin Lesions Following Antibiotic Treatment of CTCL Patients

**FIGURE 3:**
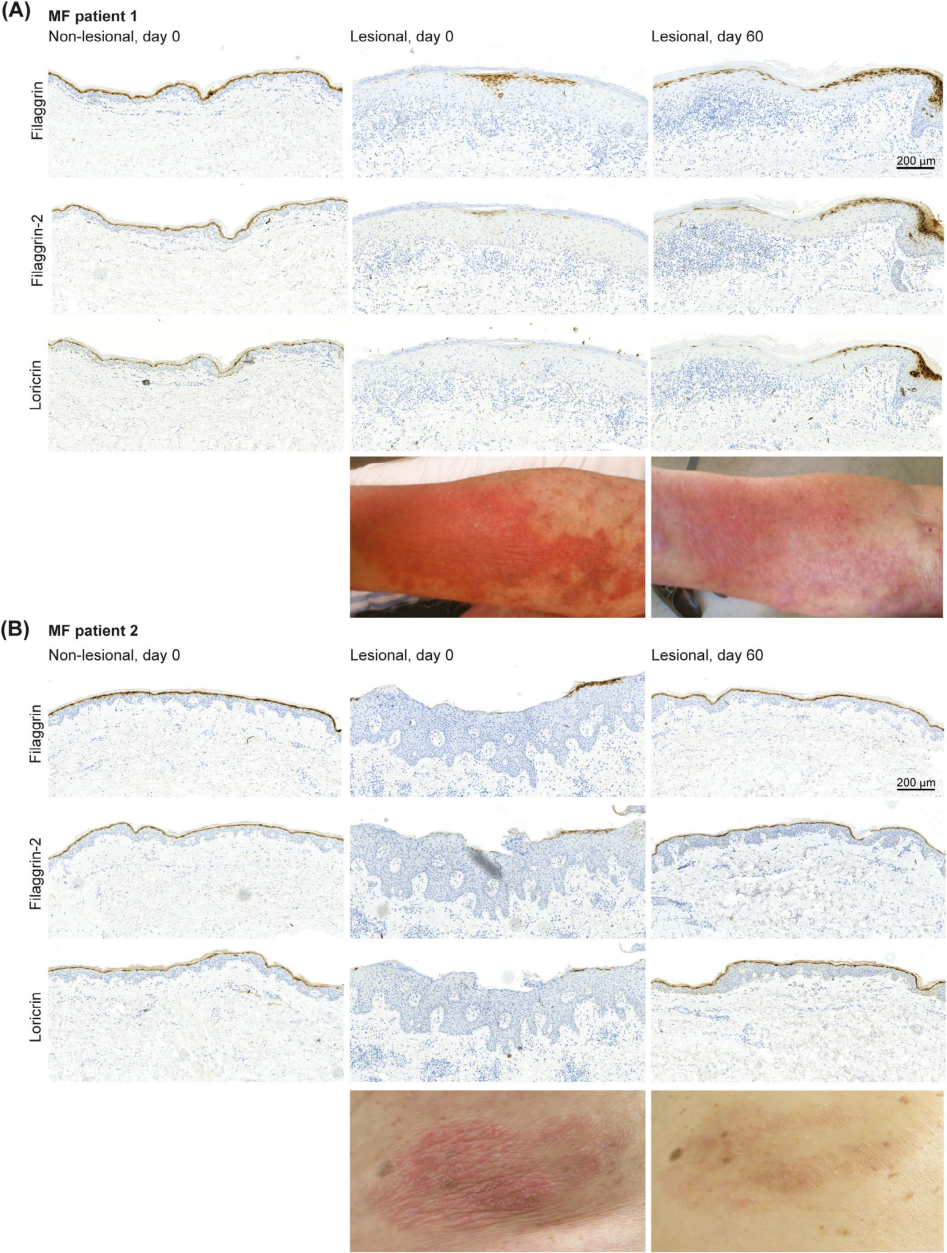
Recovery of filaggrin and loricrin expression in skin lesions following antibiotic treatment of CTCL patients. (A, B) IHC analysis of lesional and non‐lesional skin site biopsies obtained from two MF patients (A: MF1, B: MF2) before initiation of antibiotic treatment (Day 0) and after treatment (Day 60). Samples were stained for filaggrin, filaggrin‐2, and loricrin. IHC images were scanned by the Zeiss Axio Scan.Z1 in 20×, and a size bar of 200 μm is included.

As antibiotics were previously shown to decrease malignant T cells in CTCL skin lesions colonised by SE‐producing *S. aureus* [35], we investigated the expression of filaggrins and loricrin in skin lesions before (Day 0) and 2 months (60 days) after initiation of 24‐days of antibiotic treatment to eliminate SE‐producing *S. aureus* in four advanced stage MF patients [35]. In an attempt to control for confounding factors, CTCL‐directed anticancer therapies remained unchanged from 2 months before inclusion and during the study. Figure 3 shows filaggrin, filaggrin‐2 and loricrin‐ expression in lesional and non‐lesional skin in two MF patients (A: MF1 and B: MF2) before (Day 0) and after (Day 60) initiation of antibiotic treatment [35]. Large areas of deficient filaggrin, filaggrin‐2, and loricrin protein expression were observed in lesional skin prior to treatment when compared to non‐lesional skin (Figure 3). Expression of filaggrin/loricrin‐ proteins increased after initiation of antibiotic treatment (Day 60), when compared to the levels prior to antibiotics (Figure 3, Day 0). The increase in filaggrin and loricrin concurred with clinical improvement (Figure 3) and a previously reported improvement in clinical scores; subjective symptoms (VAS) and mSWAT (Table S4) [35]. Similar findings were obtained for another patient (MF3) (Figure S3A). In contrast, one patient (MF4) did not display an increase in filaggrin/loricrin expression at Day 60, but localised areas of decreased expression seemed to be more pronounced in this patient (Figure S3B). Likewise, mSWAT was higher in this patient at day 0 compared to patient MF1‐MF3, indicating high disease burden (Table S4). The analytical Histoscore was applied to evaluate the expression of filaggrins and loricrin throughout the full sections of all four patients (Figure S3C) and confirmed that expression of filaggrins and loricrin was increased after antibiotic treatment in three patients MF1‐MF3, but not in patient MF4. Figure S3D shows gene expression microarray (Affymetrix) data obtained from analysis of lesional and non‐lesional skin from eight patients (six MF and two SS) [35] prior to (Day 0), during (Day 10), and after antibiotic treatment (Day 30). At Day 0, the expression of FLG, FLG2, and LOR was markedly lower in skin lesions compared to non‐lesional skin which was in accordance with previous reports [26, 27]. After initiation of antibiotic treatment, we observed an increase in FLG, FLG2 and LOR expression in lesional skin whereas non‐lesional skin was largely unaffected (Figure S3D).

### 3.5 The Presence of Immune Infiltrates Correlates With Reduced Expression of Filaggrin

**FIGURE 4:**
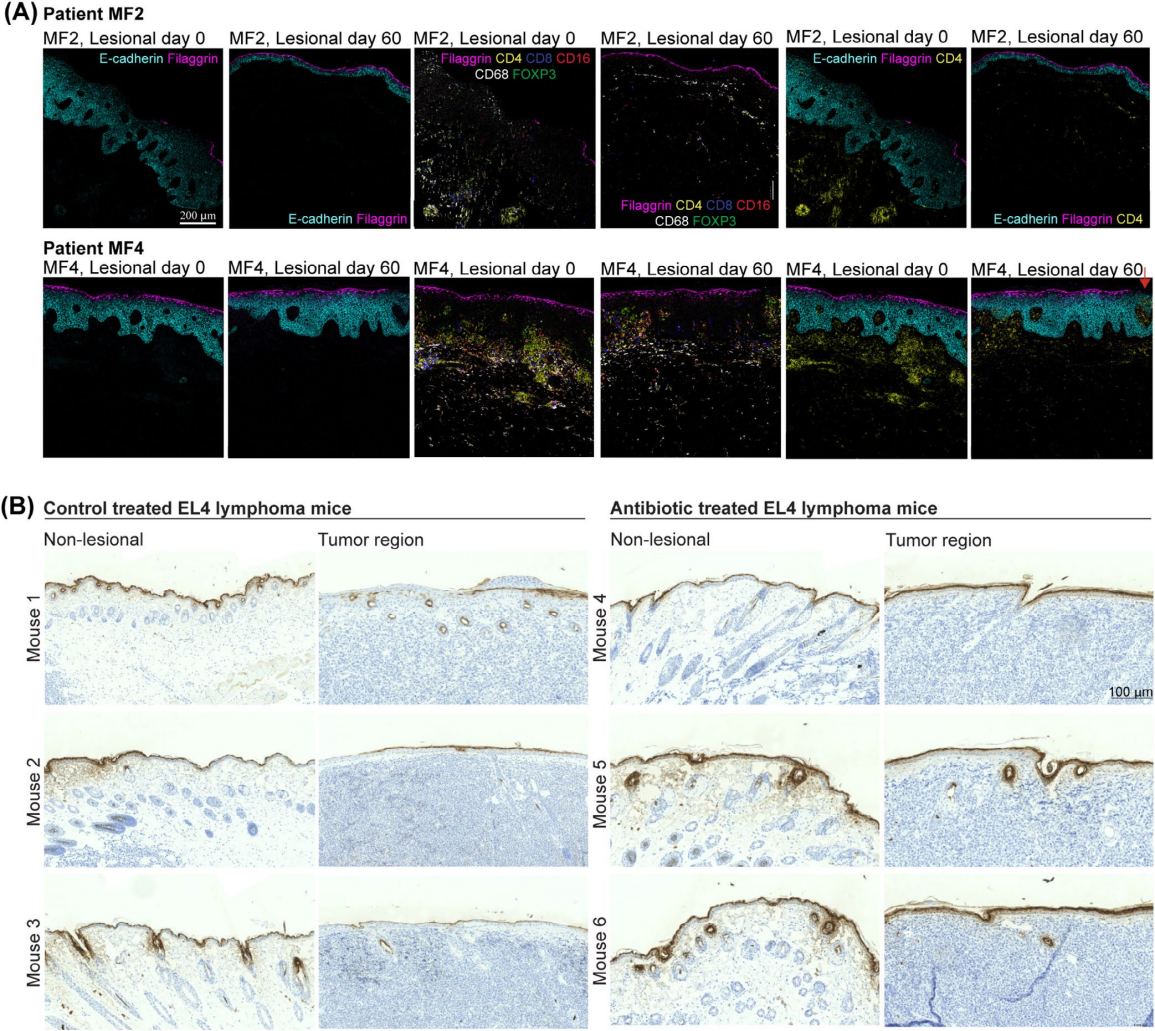
Recovery of filaggrin analysed using Imaging mass cytometry and the EL4 mouse model. (A) Imaging mass cytometry (Hyperion) analysis of lesional skin biopsies of two patients MF2 and MF4 before and after antibiotic treatment. Samples were stained for filaggrin (magenta), E‐cadherin (turquoise), CD4^+^ (yellow), CD8^+^ (blue), CD16^+^ (red) CD68^+^ (white) and FOXP3^+^ (green) and images were scanned using imaging mass cytometry (Hyperion). The software MCD viewer was applied to analyse the data and a size bar of 200 μm is included in the images. (B) IHC analysis of non‐lesional and lesional (tumour‐region) skin site biopsies obtained from six mice inoculated with EL4 cells—three mice treated with Vaseline (mouse 1,2,3) and three mice treated with Neosporin (mouse 4,5,6). Samples were stained for filaggrin and IHC images were scanned by the Zeiss Axio Scan.Z1 in 20× and a size bar of 100 μm is included.

To analyse the immune infiltrates in lesional skin before and after antibiotic treatment imaging mass cytometry was applied. Figure 4A and Figure S4A show protein expression of filaggrin (magenta), E‐cadherin (turquoise), CD4^+^ T cells (yellow), CD8^+^ T cells (blue), CD16^+^ (red), CD68^+^ (white), FOXP3^+^ T cells (green), at Day 0 and Day 60 in patients MF2, MF3, MF4, where tissue sections were still available. The expression pattern of filaggrin was very similar to the expression patterns obtained from IHC analysis (Figure 4A vs. Figure 3; Figure S4A,B). Increased filaggrin was observed after initiation of antibiotic treatment when compared to Day 0 in patients MF2 and MF3 (Figure 4; Figure S4A), whereas patient MF4 had relatively unchanged filaggrin expression at Day 60 confirming the above findings in IHC (Figure 4 vs. Figure S3B). Interestingly, the improvement in filaggrin expression in patients MF2 and MF3 was associated with a decrease in immune infiltrates and in particular CD4^+^ T cells (Figure 4; Figure S4A,B). An overall drop in CD4^+^ T cells was also observed at Day 60, but in contrast to patients MF2 and MF3, patient MF4 displayed a large intra‐epidermal infiltrate of CD4^+^ T cells adjacent to an area of the epidermis with an almost complete absence of filaggrin staining (Figure 4, arrow).

### 3.6 Antibiotic Treatment Improves Filaggrin Expression in the Murine EL4 T Cell Lymphoma Model

The EL4 murine T cell lymphoma model of bacteria‐driven tumour progression was used to assess epidermal filaggrin expression in skin from mice that were treated with and without antibiotics as described elsewhere [51]. Six mice were inoculated intradermally with EL4 cells, and three were treated with antibiotics (Neosporin (neomycin/bacitracin/polymyxin B sulphate)), and three mice treated with vehicle control (Vaseline) [51]. Filaggrin expression was compromised in skin adjacent to tumour areas (when compared to non‐lesional skin) in all the three vehicle control mice (Figure 4B, left), whereas filaggrin expression was near‐normal in keratinocytes in skin areas adjacent to tumour cells in the antibiotic treated mice (Figure 4B, right). A similar difference was observed when assessing the overall histoscores on keratinocyte expression of filaggrin (Figure S4C). In contrast, antibiotics had no effect on the baseline expression of filaggrin in skin from healthy control mice (Figure S4D).

### 3.7 Decreased STAT1, STAT3 and STAT6 Activation After Antibiotic Treatment

**FIGURE 5:**
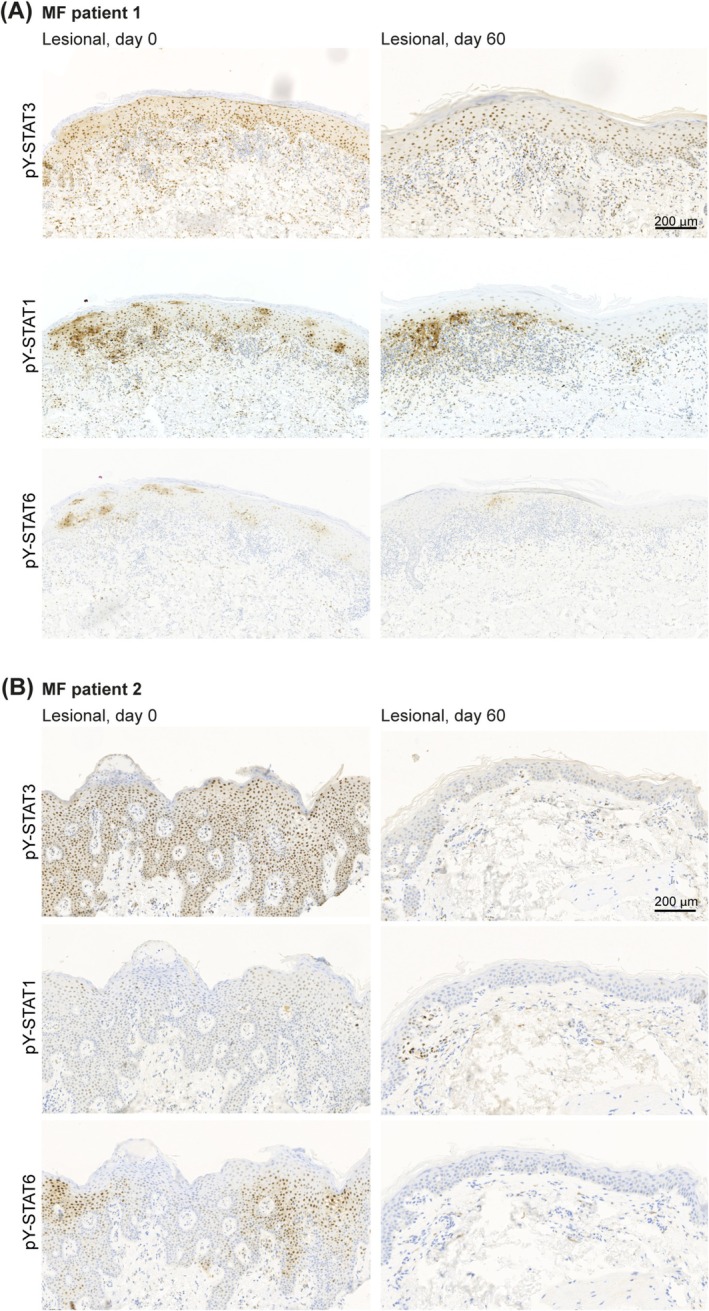
Decreased STAT1, STAT3 and STAT6 activation after antibiotic treatment. (A, B) IHC analysis of lesional skin site biopsies obtained before (Day 0) and after (Day 60) initiation of antibiotic treatment from two MF patients (A: MF1 and B: MF2). Samples were stained for pY‐STAT1, pY‐STAT3 and pY‐STAT6. Images were scanned by the Zeiss Axio Scan.Z1 in 20× and a size bar of 200 μm is included.

Lesional skin biopsies from patients MF1‐MF4 were stained for activated (pY)‐STAT3, pY‐STAT1 and pY‐STAT6. IHC analysis demonstrated strong positive pY‐STAT3 staining in the tumour microenvironment, that is, in both dermal and epidermal layers of lesional skin (Figure 5; Figure S5A,B), whereas the corresponding non‐lesional skin stained negative as previously published (Figure S5C) [26]. Two of four patients (MF1 and MF4) stained positive for epidermal pY‐STAT1 as well as areas of pY‐STAT1 in immune infiltrates but not in non‐lesional keratinocytes (Figure 5; Figure S5A–C). pY‐STAT1 staining was predominantly positive in local areas as compared to pY‐STAT3 staining which was broadly distributed. In three of four patients (MF1, MF2, MF4) local pY‐STAT6 expression was observed, varying from very local staining to a broader staining pattern (Figure 5; Figure S5A,B). The four patients differed in intensity and staining patterns before and after initiation of antibiotics but overall STAT1, STAT3, and STAT6 activation decreased from Day 0 to Day 60 in patients (MF1‐MF3), which displayed a concomitant increase in filaggrin/loricrin expression. In contrast, a clear decrease in STAT activation was not observed in patient MF4. On the contrary, pY‐STAT1 and pY‐STAT6 staining was increased locally in some areas of the epidermis in this patient and pY‐STAT3 staining was increased as visualised by the histoscore (Figure S5B; Table S4) suggesting a possible explanation why the filaggrin/loricrin expression remained compromised at Day 60 in this patient.

### 3.8 Upstream Cytokine Signalling Decreases After Antibiotic Treatment

**FIGURE 6:**
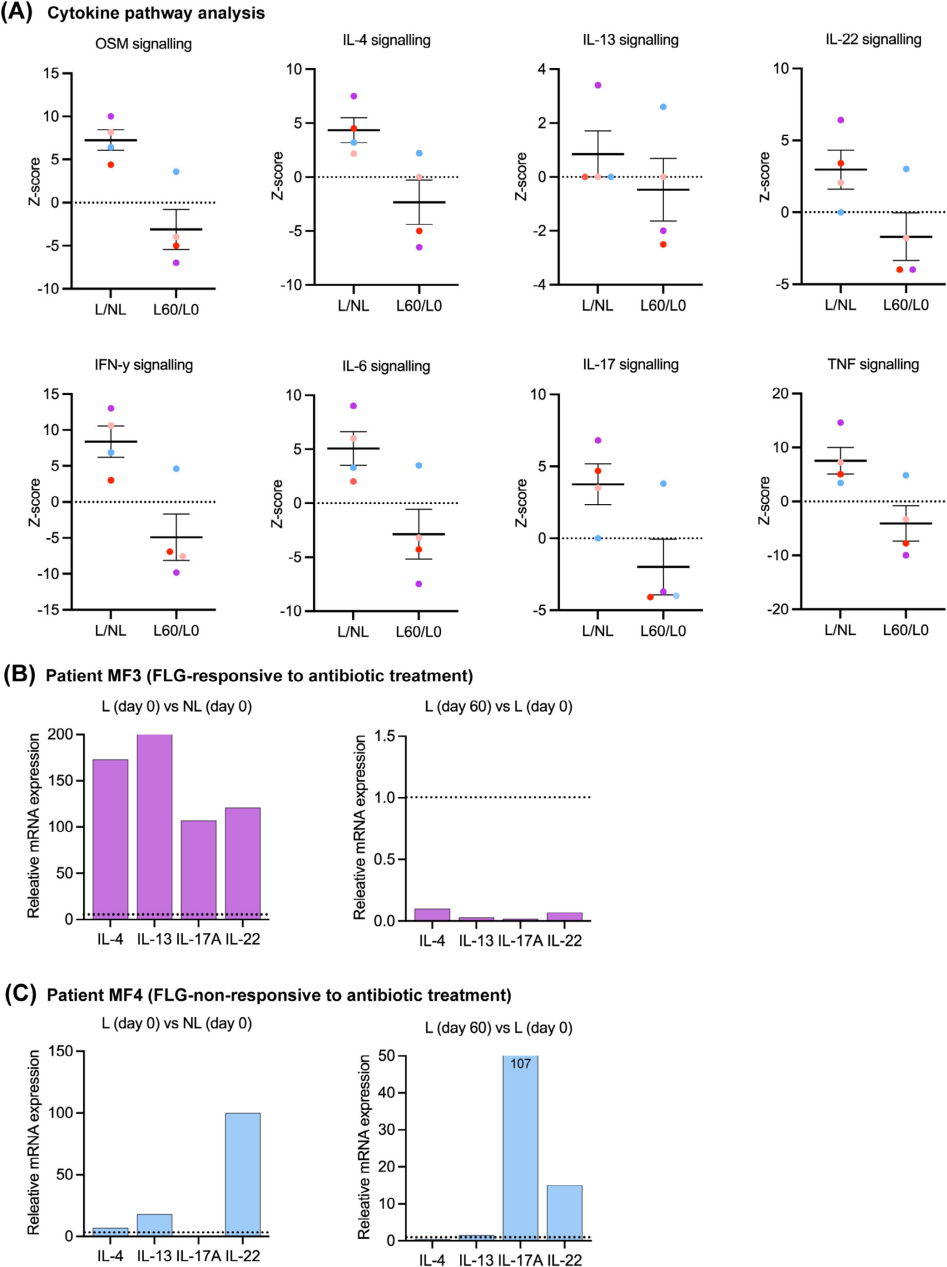
Upstream cytokine signalling decreases after antibiotic treatment. (A) Upstream cytokine pathway analysis based on gene enrichment was studied for the cytokines IL‐4, IL‐6, IL‐13, IL‐17, IL‐22, IFN‐γ, OSM and TNF‐α in lesional compared to non‐lesional skin samples as well as in lesional sites following antibiotic treatment. Transcriptomic data from samples obtained before (Day 0) and after treatment (Day 60) with antibiotics from patient MF1 (pink), MF2 (red), MF3 (purple) and MF4 (blue) were studied. The Regulator Analysis in IPA was applied and the activation state was predicted as activated for z‐score > 2 (activation of upstream cytokine signalling), and predicted as inhibited for z‐score < −2. (B) mRNA expression analysis of IL‐4, IL‐13, IL‐17 and IL‐22 in skin samples from MF3 (purple) and (C) MF4 (blue) using GAPDH as a reference gene. mRNA from lesional samples from Day 0 was first compared to NL samples and then mRNA from lesional samples Day 0 were compared to lesional Day 60. L, lesional; NL, non‐lesional.

Next, we further reanalysed gene expression microarray (Affymetrix) data from the skin lesions (MF1‐MF4) obtained before and 60 days after initiation of antibiotics [35] by performing upstream signalling pathway analysis for cytokines (due to a low content of specific cytokine mRNA). Upstream signalling was highly activated at Day 0 in lesional compared to non‐lesional skin samples from all four patients (Figure 6A). Interestingly, in three patients (MF1 (pink), MF2 (red), MF3 (purple)) lower z‐activation scores were detected after antibiotics (Figure 6A) whereas the values remained largely unchanged at Day 60 in patient MF4 (blue, Figure 6A). In two patients (MF3 and MF4), we were able to obtain enough mRNA to perform RT‐qPCR analysis for IL‐4, IL‐13, IL‐17A and IL‐22, which showed a similar pattern of cytokine expression as indicated by the analysis above (Figure 6B,C). Thus, the presence of cytokine signalling after antibiotic treatment is consistent with the lack of filaggrin/loricrin improvement in patient MF4.

## 4 Discussion

Here we provide the first evidence that SE‐producing *S. aureus* can exacerbate skin barrier defects in CTCL by inducing filaggrin/loricrin‐repressive cytokines from CTCL cells, and, inversely, that bacterial elimination is associated with an improved expression of skin barrier proteins in lesional skin in three of four investigated CTCL patients. SE‐producing *S. aureus* isolated from MF and SS patients stimulated malignant T cells to secrete factors that suppressed filaggrin expression in keratinocytes in vitro—an effect reversed by antibiotics or *S. aureus* targeting endolysins. Notably, purified SEs fully replicated the impact of *S. aureus* supernatants.

Upon exposure to SEs both malignant and non‐malignant CTCL cells displayed a cytokine–expression profile resembling that seen in skin lesions [26, 30, 48, 67, 68]. Accordingly, we hypothesise that SE‐stimulation may replicate (some of) the stimuli that malignant T cells encounter in the tumour microenvironment in lesional skin. Indeed, although we used an extracutaneous source of malignant T cells for the setups, it was highly remarkable that SEs triggered expression of a series of cytokines, which are expressed in skin lesions from CTCL patients. Of notice, IL‐13, IL‐17, IL‐22, IFN‐γ and OSM (which were all induced by SEs) have previously been detected in situ in CTCL skin lesions and linked to filaggrin repression in CTCL and/or inflammatory skin diseases. These cytokines, previously linked to JAK/STAT‐mediated filaggrin repression in CTCL and inflammatory skin diseases, mirrored the cytokine profile observed in CTCL lesions [26, 30, 31, 66, 67, 68, 69, 70, 71] and JAK inhibition largely abrogated SE‐induced repression of filaggrins and loricrin in keratinocytes supporting the notion above that *S. aureus* ‐derived SEs drive a cytokine‐mediated, JAK/STAT‐dependent repression of filaggrin and loricrin in CTCL.

In a previous clinical pilot study, antibiotic treatment reduced disease activity and the proportion of malignant T cells in situ in patients with SE‐producing *S. aureus* colonisation [35]. In four patients sampled before (Day 0) and after intensive antibiotic treatment (Day 60) [35] (concomitant CTCL‐directed treatment remained unchanged), three patients (MF1‐MF3) showed increased filaggrin, filaggrin‐2, and loricrin expression at Day 60, alongside with reduced STAT1, STAT3, and/or STAT6 activation and decreased cytokine activity (based on upstream pathway analysis of gene expression microarray data) [35]. In contrast, patient MF4 did not exhibit increased filaggrin/loricrin expression post‐treatment and persistent CD4^+^ epidermotropic T‐cell infiltrates were observed, suggesting that malignant T cells (independent of *S. aureus* ) may drive filaggrin suppression in this patient. Consistently, a previous study reported skin barrier defects near CD4^+^ T‐cell infiltrates, even in patients without *S. aureus* colonisation [26]. Moreover, the non‐responsive patient (MF4) had a higher mSWAT value at Day 0 compared to the responsive patients (MF1‐MF3 patients) indicating a more severe disease burden in patient MF4 prior to antibiotic treatment. Indeed, at Day 60, the mSWAT value for MF4 was still high compared to MF1‐MF3. Reversely, STAT3 activity (pY‐STAT3) increased from Day 0 to Day 60 in MF4 whereas it dropped in MF1‐MF3. Taken together, these findings suggest that patient MF4 displayed a more severe disease burden at start and has higher levels of pY‐STAT3 after antibiotic treatment, which aligns with a continued filaggrin/loricrin deficiency at Day 60.

The improved filaggrin expression following antibiotic treatment in the EL4 tumour mouse model aligned with our preliminary clinical findings supporting our hypothesis that *S. aureus* exacerbates CTCL‐driven skin barrier dysfunction. As we observe inter‐individual viability, larger cohorts of SS‐ and MF‐patients are needed to substantiate this hypothesis and if they do, treatment and prevention of *S. aureus* colonisation could represent a key adjuvant strategy (together with tumour‐targeting treatments) to restore skin barrier integrity in CTCL.

In conclusion, we identify a mechanism in which *S. aureus* drives cytokine production leading to skin barrier damage. Our findings suggest that eradicating *S. aureus* reduces SE‐driven inflammation and partially reverses barrier impairment. This supports the existence of a vicious cycle in which SE‐producing *S. aureus* amplifies T‐cell activation, further weakens the barrier, and promotes renewed colonisation. Breaking this cycle may require combined strategies targeting malignant T cells, the inflammatory microenvironment, and selective anti‐bacterial approaches.

## Author Contributions

Concept and design: M.G., T.B.B., and N.Ø. Acquisition, analysis, or interpretation of data: All authors. Drafting of the manuscript: M.G. and N.Ø. Critical revision of the manuscript for important intellectual content: All authors. Obtained funding: N.Ø. Administrative, technical, or material support: M.G., E.P., C.K.V., M.N., Z.Z., L.M.R.G., L.Y., S.A., L.M.L., M.B., M.R.K., S.D., S.R.O., E.S., P.R., C.M.B., C.G., A.W., R.L., L.I., P.W., T.L., T.B.B., N.Ø. Supervision: T.B.B. and N.Ø.

## Funding

This research was funded by LEO Foundation through the LEO Foundation Skin Immunology Research Center, Dr. Abildgaard Fellowship (LF‐FE‐23‐700015; T.B.B.), the Danish Cancer Society (Kræftens Bekæmpelse; C.K.V. and N.Ø.) the Fight Cancer Program (Knæk Cancer; R132‐A8475; NØ) and donation from Fabrikant Vilhelm Pedersen og Hustrus Legat (Kræftens Bekæmpelse R374‐A22434); Novo Nordisk Research Foundation (N.Ø.), Novo Nordisk Foundation Tandem Program—NNF210C0066950 in the call “Tandem Programme 2021” (N.Ø.), the Danish Council for Independent Research (Danmarks Frie Forskningsfond, grant number: 0134‐00385B and grant https://doi.org/10.46540/3165‐00211B; N.Ø.). RL was funded by Reserach Council of Finland grant 331793 and The Sigrid Jusélius Foundation. Role of the funding source: The funding source had no influence on the design and conduct of the study; collection, management, analysis, and interpretation of the data; preparation, review, or approval of the manuscript; and decision to submit the manuscript for publication.

## Conflicts of Interest

Niels Ødum has received consulting honoraria from Micreos Human Health. Lise Mette Rahbek Gjerdrum has received consulting fees from Kyowa Kirin. Thomas Litman is funded by LEO Pharma. The remaining authors declare no conflicts of interest.

## Supporting information


**Data S1:** all70292‐sup‐0001‐Supinfo.pdf.

## Data Availability

Previously published Single‐cell RNA‐seq data and microarray data are available from Gene Expression Omnibus at accesssions GSE284075 and GSE122934, respectively.
