# Supplementary material for: Staphylococcus aureus Augments Epithelial Skin Barrier Damage Through T Cell Activation in Cutaneous T Cell Lymphoma
Source: Allergy. 2026 Mar 8;81(7):2435–49. doi: 10.1111/all.70292 (PMC13342767; doi:10.1111/all.70292)

Supplemental material

Supplemental Tables

Supplemental Table 1: Patient characteristics

Patient	Stage	Treatment*	SA/SE detection
MF1	IIIA	Topical corticosteroids	+/SEA
MF2	IIB	NM and topical steroids	+/SEA
MF3	IIB	Acretin and topical corticosteroids	+/SEA
MF4	IIIA	Topical steroids	+/SEA

*CTCL-directed anticancer therapy and dosing remained unchanged from at least 2 months before inclusion and not changed during the study.
* NM: nitrogen mustard

Supplemental Table 2: Murine EL4 lymphoma

ID	Mouse	Location	Treatment	Sacrifice day
Mouse 7	Mouse C57/BL6	Skin	Vaseline	Day 30
Mouse 3	Mouse C57/BL6	Skin: tumor + healthy	Vaseline	Day 20
Mouse 1	Mouse C57/BL6	Skin: tumor + healthy	Vaseline	Day 19
Mouse 2	Mouse C57/BL6	Skin: tumor + healthy	Vaseline	Day 21
Mouse 8	Mouse C57/BL6	Skin	Neosporin	Day 30
Mouse 6	Mouse C57/BL6	Skin: tumor + healthy	Neosporin	Day 27
Mouse 5	Mouse C57/BL6	Skin: tumor + healthy	Neosporin	Day 27
Mouse 4	Mouse C57/BL6	Skin: tumor + healthy	Neosporin	Day 28

Supplemental Table 3: List of antibodies used for Hyperion

Antibody	Clone	Metal-conjugation	Concentration	Manufacturer
Nuclei cell ID	-	-	1:200	Flugidigm
Filaggrin	FLG01	-	1:50	GeneTex
E-cadherin	24E10	174Yb	1:100	Flugidigm
Vimentin	D21H3	143Nd	1:200	Flugidigm
Pan-cytokeratin	C11	148Nd	1:200	Flugidigm
FOXP3	PCH101	155Gd	1:50	Flugidigm
CD3	UCHT1	170Er	1:200	Flugidigm
CD4	EPR6855	156Gd	1:100	Flugidigm
CD8	C8/144B	162Dy	1:100	Flugidigm
CD16	EPR16784	146Nd	1:50	Flugidigm
CD45	D9M8I	152Sm	1:100	Flugidigm
CD68	KP1	159Tb	1:50	Flugidigm
Goat Anti-mouse	-	172Yb	1:200	Flugidigm

Supplemental Table 4: Patient details

Patient	mSWAT day 0	mSWAT day 60	SE day 0	H-score* (pY-STAT3) day 0	H-score* (pY-STAT3) day 60
MF1	110	90	SEA, SEJ	107	85
MF2	25	10	SEA	83	4
MF3	110	50	SEA, SEJ	104	79
MF4	160	135	SEA	86	124

*Histoscore of epidermal pY-STAT3

Supplemental methods

Staphylococcus aureus (*S. aureus*) cultures

S. aureus cultures isolated from CTCL patiens were grown in TSB medium with or without antibiotics (meropenem, 8µg/mL) or 1 µg/mL of the S. *aureus* targeting compounds Endolysin XZ700 or MEndoB or the MEndoB-mutant (Micreos Group, The Netherlands). ELISA (Ridascreen set, R-Biopharm) was applied to detect the presence of enterotoxins in the bacterial supernatant; SA.1 (+SEA, +SEE), SA.2 (+SEA), SA.3 (-).

Keratinocyte experiments

Normal human epidermal keratinocytes (NHEK) were stimulated directly with bacterial supernatants from SA.1 – one part to three parts of keratinocyte media.

Reconstructed human epidermis

Reconstructed human epidermis (RHE) models (Episkin, SkinEthic RHE, S-17, 0.5 cm^2) were cultured according to the manufacturer's instructions. RHE samples were cultured for 48h with control medium, or supernatants obtained from CTCL cells stimulated with the SE-pool for 48h.

CITE-sequencing

Previously published CITE-seq data sets were reanalysed for cytokine expression. Patients SS1-SS6 were previously noted as SS1: PT03, SS2: PT12, SS3: PT19, SS4: PT11, SS5: PT20, SS6: PT13[33-34]. CITE-seq staining protocol and antibodies were individually optimised and titrated. Following four rounds of washing by centrifugation, cells were counted and loaded into a single reaction well on a 10X Chromium Chip K following manufacturer's instructions (10X Genomics). After cDNA synthesis, emulsion recovery and clean up, full length cDNA was amplified by 13 cycles of PCR with addition of 25 nM of primers targeting the PCR handle present on all the hashtag oligo (HTO) antibodies (HTO_add: GTGACTGGAGTTCAGACGTGTGC*T*C) and antibody-derived tags (ADT; TotalSeq-C) antibodies (ADT_add: CTCGTGGGCTCGGAGATGTGTATAAGAG*A*C) together with the cDNA primers included in the Chromium Next GEM Single Cell 5' reagent kit v2 (10X Genomics). Following size-selection, HTO and ADT sequencing libraries were constructed separately from the small cDNA fraction by 9 and 12 cycles of PCR using TruSeq/TruSeq and TruSeq/Nextera P5/P7 indexing

primers, respectively. Gene expression (GEX) and TCRαβ sequencing libraries were constructed from the large cDNA fraction following manufacturer's instructions. HTO, ADT, GEX and TCRαβ libraries from batches of multiple SS patients were sequencing together on an Illumina NovaSeq6000 S4 flow cell. Sequencing reads from HTO and ADT libraries were counted using the Kallisto-KITE workflow [https://github.com/pachterlab/kite]. Sequencing reads from GEX and TCRαβ libraries were aligned and assembled using the CellRanger (v7.0.0). Ambient signal from GEX, ADT and HTO libraries was removed using CellBender. Initially cell barcodes having less than 100 genes detected or more than 50% of unique molecular identifiers (UMIs) derived from mitochondrial transcripts were removed. Further filtering was done following "overclustering" with scran::clusterCells clustering (NNGraphParam with k=5) and removal of clusters that consistently have low UMI counts, low number of genes detected and high fraction of UMIs derived from mitochondrial transcripts. Hashtagged cells were demultiplexed into their respective conditions and cross-sample doublets removed using the hashsolo function from the solo python library. Intra-sample doublets were removed using scDblFinder R package(34). SE-pool and PBS treated samples from different SS patient were integrated for visualisation and clustering based on both gene- and surface protein expression using TotalVI with recommended parameters and using the top 4000 variable genes (using the separate reactions as batch_key) and including cell cycle "S" and "G2M" scores from Seurat::CellCycleScoring as continuous covariates when training to model (to reduce clustering based on cell cycle status). Cell types were annotated based on their expression of distinct lineage gene expression (from the GEX modality) and surface markers (from the ADT modality).

IHC analysis

For IHC, detection of the targets was performed on the Omnis platform (Dako, Glostrup, Denmark). Briefly, deparaffination and antigen retrieval was performed by immersing slides in EnVision™ FLEX Target Retrieval Solution, High pH (Dako, # K8004) following the manufacturer's instructions. After pre-treatment, slides were incubated with the primary antibodies for 30 min and reactions were detected and visualised using EnVision™ FLEX/HRP Detection Reagents (Dako, #K8000). Depending on the host of the primary antibody, signal intensity was enhanced using either EnVision™ FLEX+ Mouse-Linker (Dako, #K8021), EnVision™ FLEX+ Rabbit-Linker (Dako, #K8009) or for pY-STAT3, both linkers were applied in combination.

Finally, sections were counterstained with Hematoxylin and mounted with Pertex. For murine IHC stainings the purified anti-filaggrin (poly19058, 1:250, Biolegend,) was applied.

Microarray analysis

Transcriptomic profiling of CTCL patient biopsies from lesional and non-lesional skin samples was performed using the Affymetrix GeneChip Human Transcriptome Array 2.0 (Rigshospitalet, Denmark) and analysed as previously described[35]. Gene expression values of FLG, FLG2 and LOR were evaluated and values given as log2AFU.

Supplemental legends

Supplemental Figure 1: *S. aureus*- and SE-stimulated CTCL cells repress filaggrin expression in keratinocytes. A: Representative flow cytometry gating strategy for one patient with malignant T cells determined based on TCRvβ18$^+$CD26$^-$CD3$^+$CD4$^+$ for the patient presented. B-C: KRT14 (B) or FLG and FLG2 (C) mRNA expression analysis of NHEK stimulated with supernatants from CTCL cells cultured with TSB media (Pt(TSB)), supernatant from patient-derived *S. aureus* (Pt(SA.1)), *S. aureus* pre-treated with either antibiotics (Pt(SA.1+Abx)), endolysin (Pt(SA+XZ700)), MendoB (Pt(SA.1+MEndoB)), or the mutated MEndoB (Pt(SA.1+MEndoB+mut)). NHEK exposed directly to SA.1 supernatant showed no significant changes in FLG, FLG2 or LOR (data not shown). D: FLG, FLG2 and LOR mRNA expression analysis of NHEK stimulated with supernatants from MF cells (Pt-MF) cultured with increasing concentrations of SEA (50 pg/ml-50 ng/ml). E-F: FLG, FLG2 and LOR mRNA expression analysis of NHEK stimulated with supernatants from MF cells (E) or SS cells (F) treated with SEpool, SEA or SEE. B-F: mRNA expression analysis using β-actin as a reference gene were given as relative expression. n=3 except D (n=1). (*) indicates statistical significance ($P<0.05$). G: IHC staining of filaggrin and filaggrin-2 of RHE samples cultured for 48h with supernatant from CTCL cells stimulated for 72h with the SE-pool. IHC images were scanned by the Zeiss Axio Scan.Z1 in 20x.

Supplemental Figure 2: Bacterial derived SEs induce expression of filaggrin-repressing cytokines in CTCL cells. A: Representative mesoscale analysis detecting the presence of cytokines in supernatants collected from PBMCs obtained from a SS patient stimulated with either PBS, SE-pool for 72h or bacterial supernatant obtained from a patient-derived bacterial isolate treated with or without MEndoB or the mutated MendoB. B: Mesoscale analysis detecting the presence of cytokines in supernatants collected from PBMCs obtained from a leukemic MF patient stimulated with PBS, SEA or the SE-pool for 72h. A-B: The values illustrated in the heatmaps were given as $\log_{10}$ pg/ml.

Supplemental Figure 3: Recovery of filaggrin and loricrin expression in skin lesions following antibiotic treatment of CTCL patients. A-B: IHC analysis of lesional- and non-lesional skin site biopsies obtained from two MF patients (A: MF3, B: MF4) before initiation of antibiotic treatment (day 0) and after treatment (day 60). Samples were stained for filaggrin, filaggrin-2 and loricrin. Arrows indicate areas of filaggrin loss. IHC images were scanned by the Zeiss Axio Scan.Z1 in 20x and a size bar of 200 μm is

included. C: Presentation of histoscores (H-scores) for cytoplasmic expression of filaggrin, filaggrin-2 and loricrin in lesional skin from MF1, MF2, MF3, MF4. D: Gene expression of FLG, FLG2 and LOR in CTCL skin samples analysed by microarray (Affymetrix GeneChip). Non-lesional (NL) and lesional (L) skin samples from eight patients obtained prior to antibiotic treatment (day 0), during (day 10) and after antibiotic treatment (day 30) were evaluated. Wilcoxon signed rank test was applied to compare groups. p-values are given in the figure.

Supplemental Figure 4: Recovery of filaggrin analysed using Imaging mass cytometry and the EL4 mouse model. A-B: Imaging mass cytometry (Hyperion) analysis of lesional skin biopsies of patients before and after antibiotic treatment. A: Patient MF3 samples were stained for filaggrin (magenta), E-cadherin (turquoise), CD4+ (yellow), CD8+ (blue), CD16+ (red) CD68+ (white) and FOXP3+ (green). B: Patient MF2, MF3 and MF4 samples were stained for filaggrin (magenta) and vimentin (white). Images were scanned using imaging mass cytometry (Hyperion). The software MCD viewer was applied to analyse the data and a size bar of 200 μm is included in the images. C: Presentation of histoscores (H-scores) for expression of filaggrin in EL4 inoculated mice skin sections from control treated (Vaseline, mouse 1-3) and antibiotic treated (Neosporin, mouse 4-6). D: IHC analysis of skin site biopsies obtained from two mice; one mouse treated with (Vaseline) and one mouse treated with Neosporin. Samples were stained for filaggrin. IHC images were scanned by the Zeiss Axio Scan.Z1 in 20x and a size bar of 100 μm is included.

Supplemental Figure 5: Decreased STAT1, STAT3 and STAT6 activation after antibiotic treatment. A-B: IHC analysis of lesional skin site biopsies obtained before (day 0) and after (day 60) initiation of antibiotic treatment from two MF patients (B: MF3 and C: MF4). C: IHC analysis of non-lesional skin site biopsies before initiation of antibiotics (day 0) from two MF patients (MF1 and MF2). Samples were stained for pY-STAT1, pY-STAT3 and pY-STAT6. Images were scanned by the Zeiss Axio Scan.Z1 in 20x and a size bar of 200 μm is included.

Supplemental Figures

Supplemental Figure 1

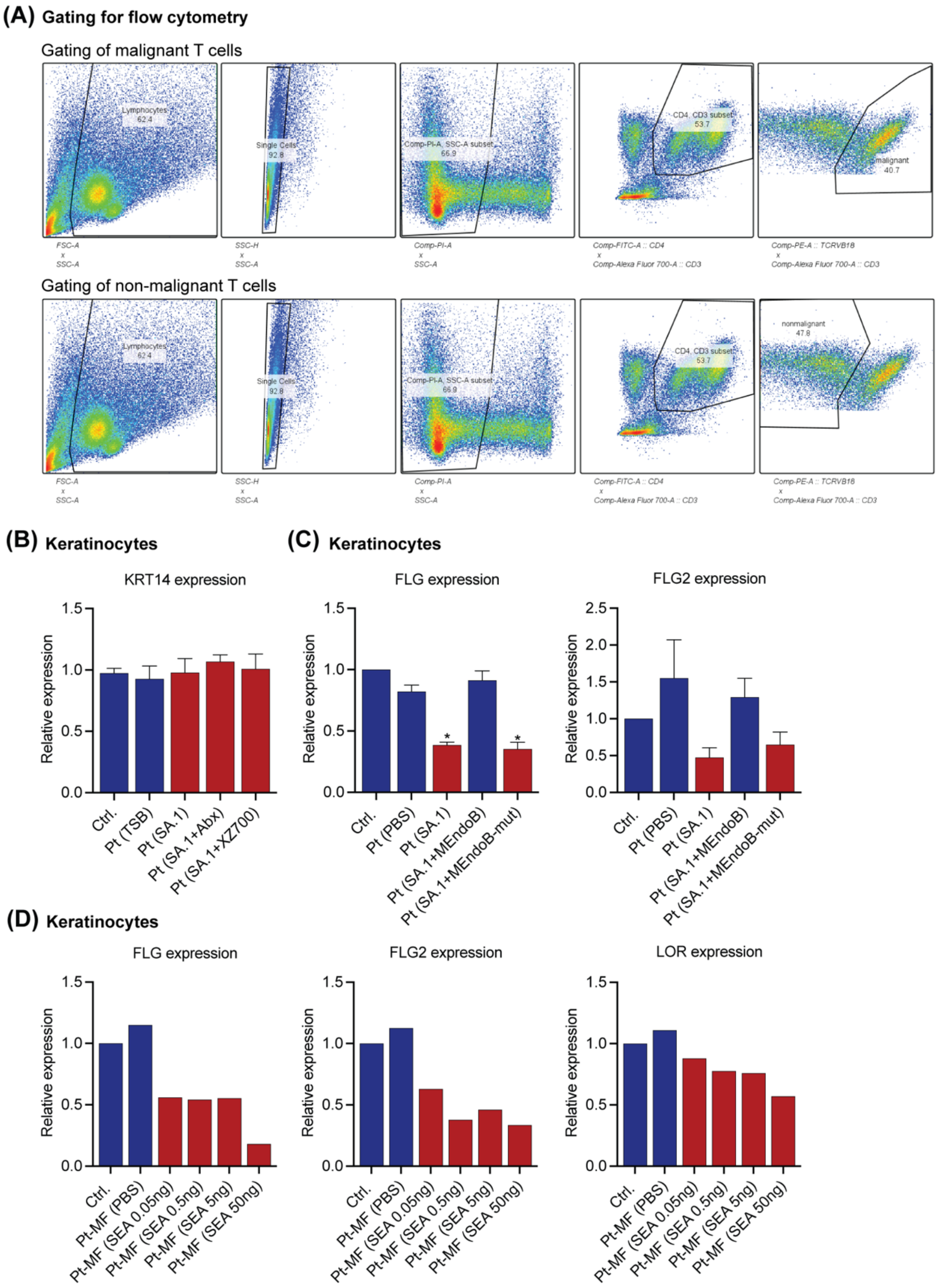

Supplemental Figure 1 *(continued)*

(E) Keratinocytes

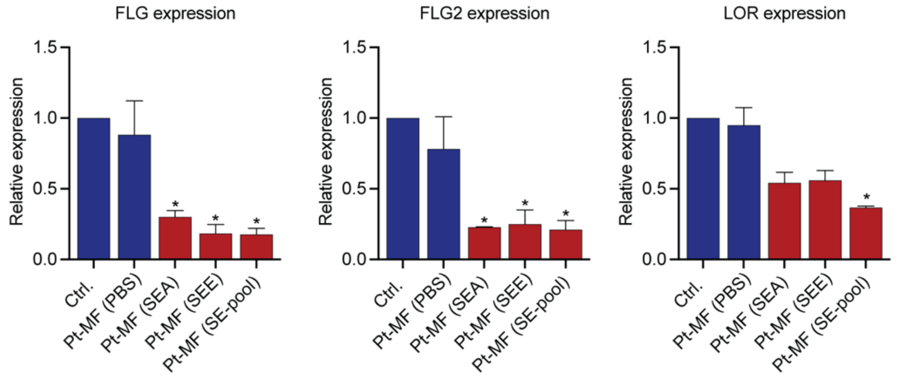

(F) Keratinocytes

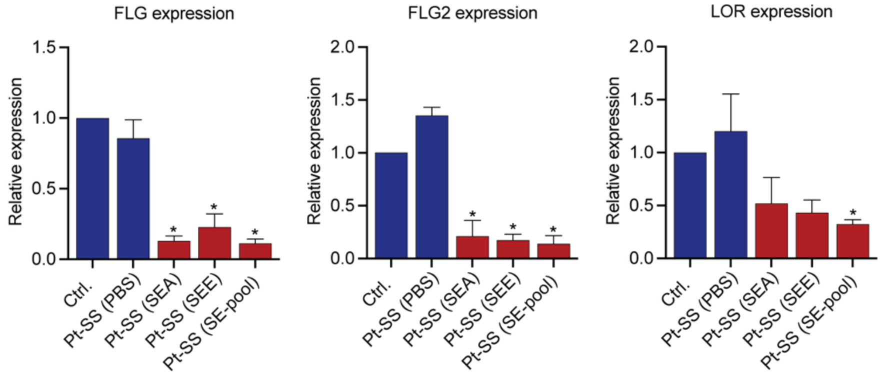

(G) Reconstructed epidermis

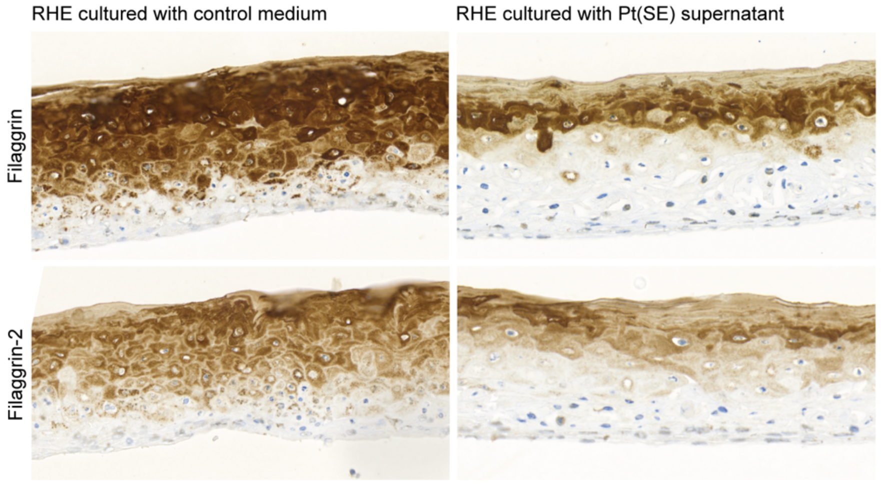

Supplemental Figure 2

(A) Heatmap

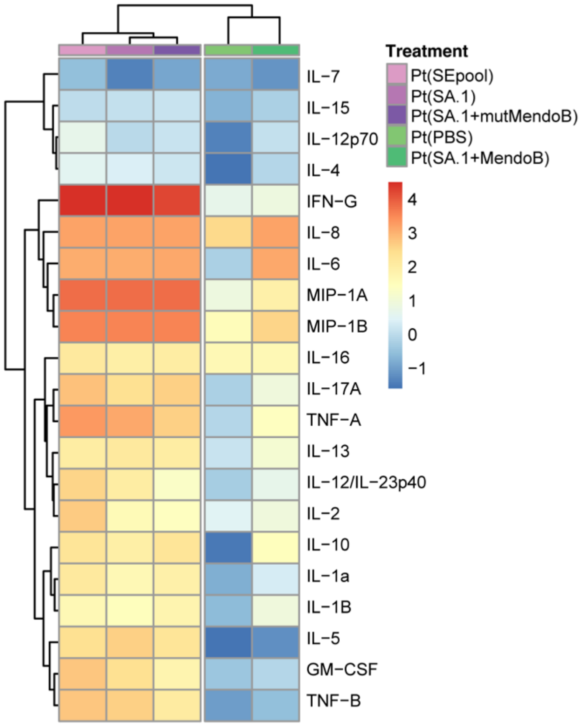

(B) Heatmap

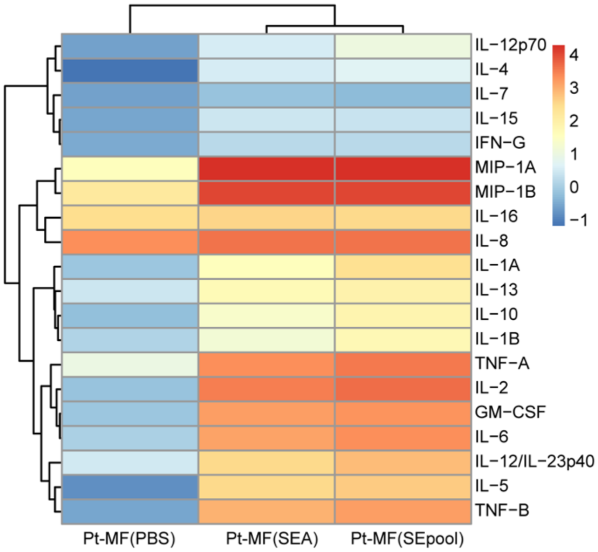

Supplemental Figure 3

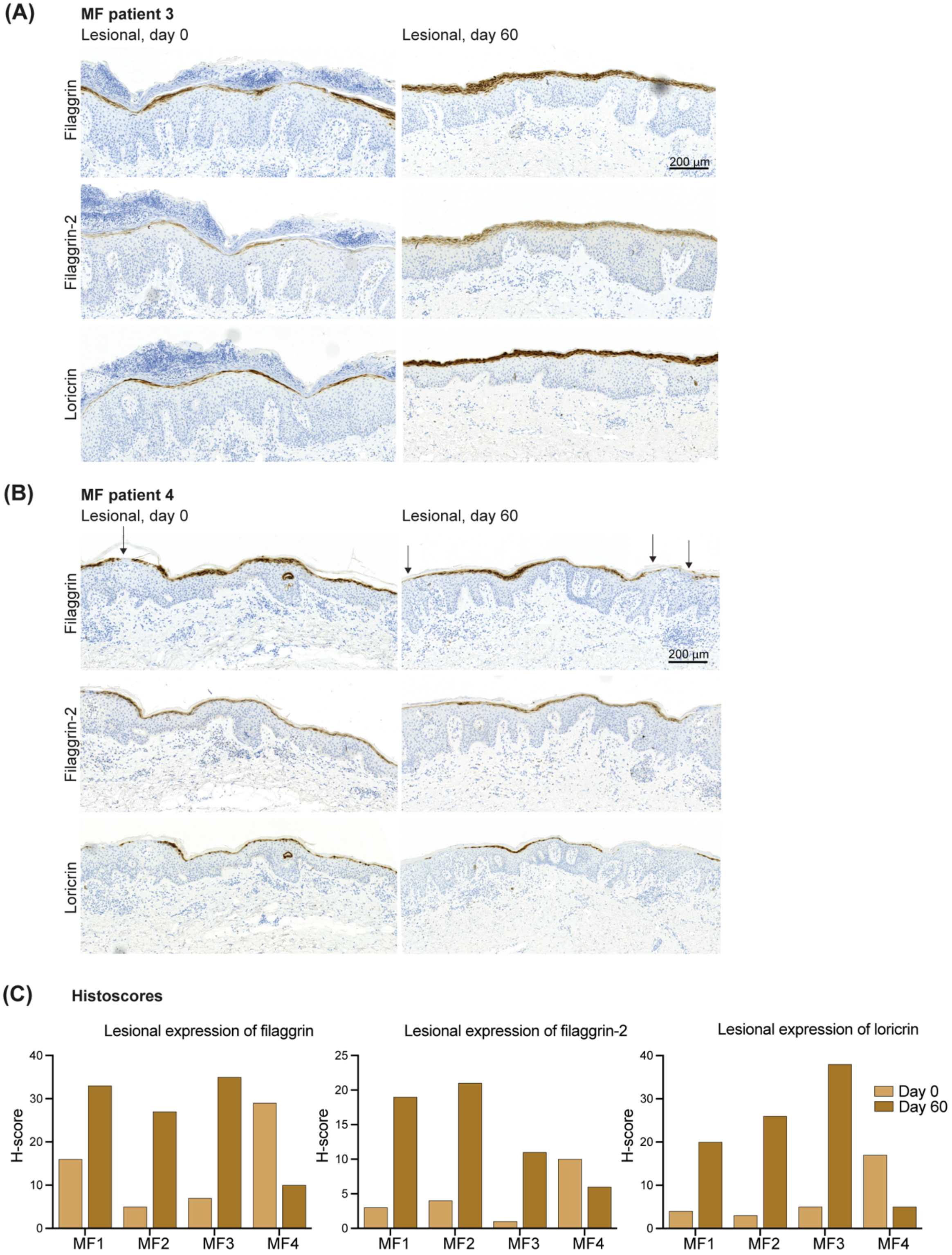

Supplemental Figure 3 *(continued)*

(D) Microarray

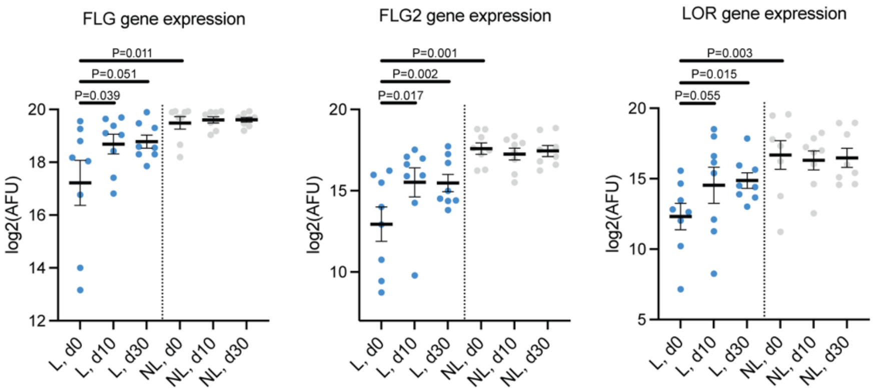

Supplemental Figure 4

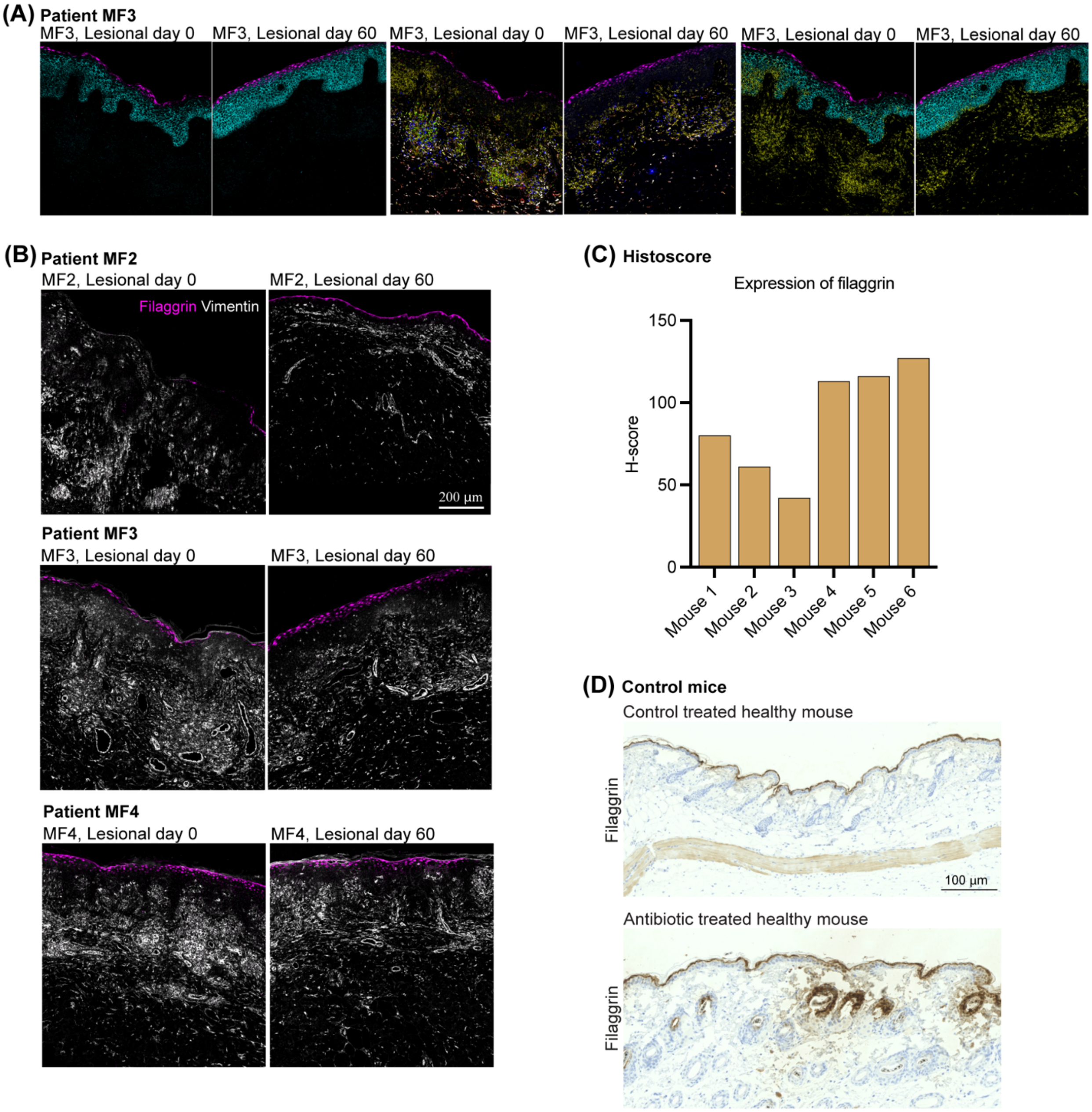

Supplemental Figure 5

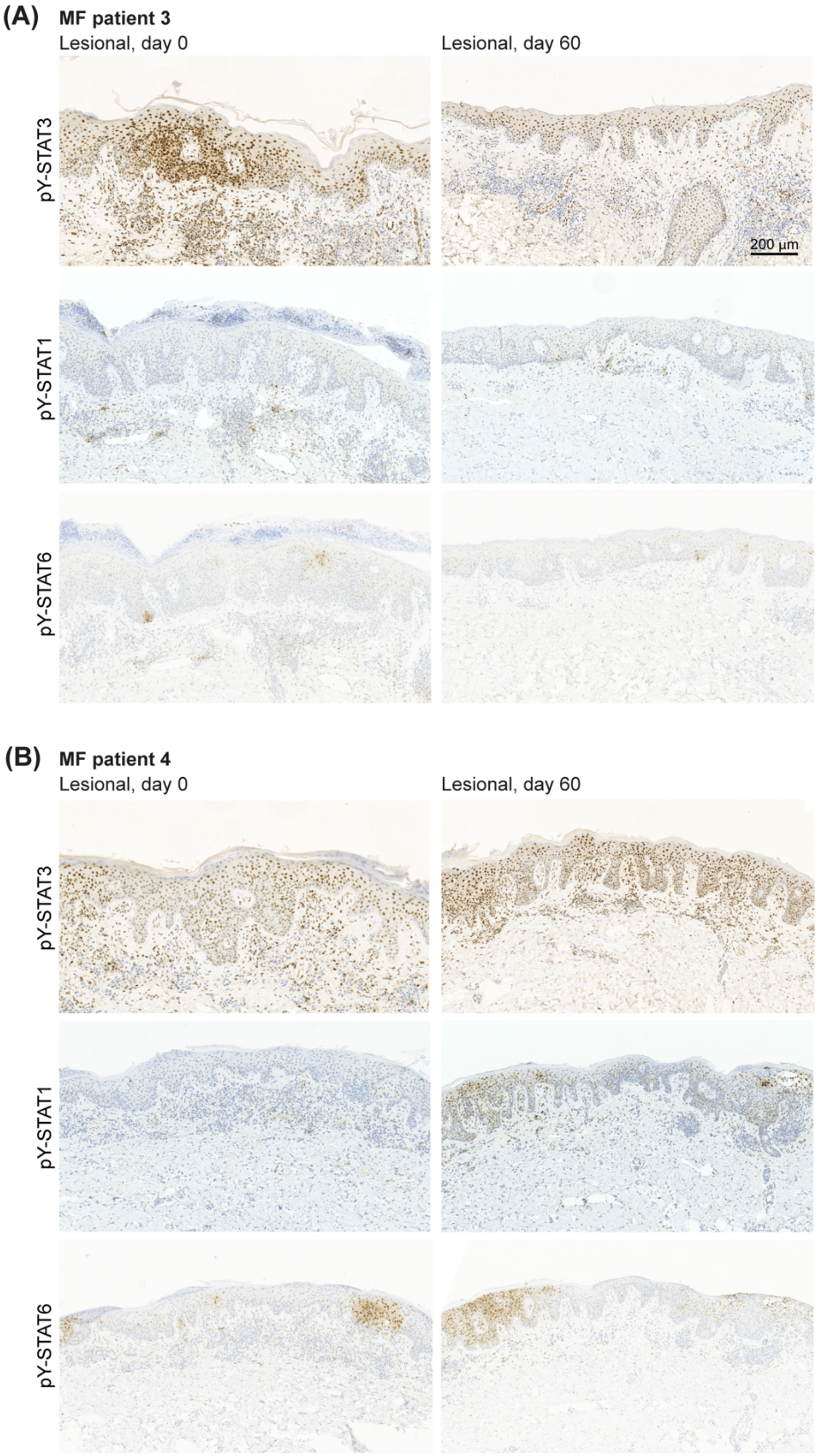

Supplemental Figure 5 *(continued)*

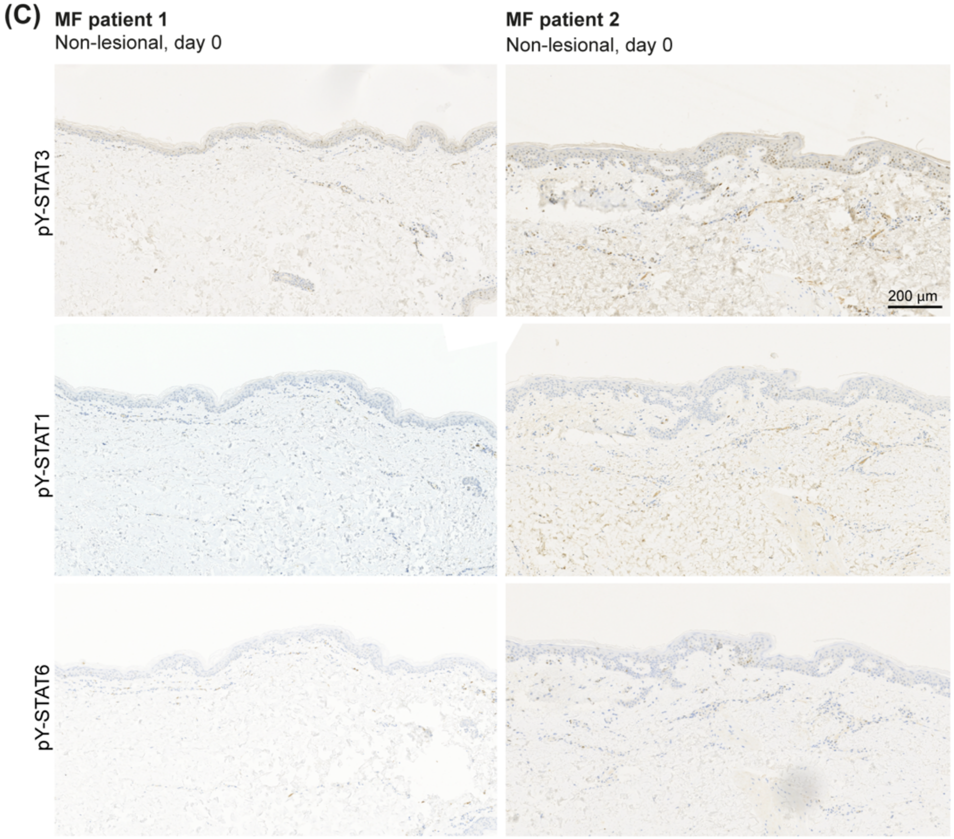

Supplement: Supplementary file 1 — Data S1: all70292‐sup‐0001‐Supinfo.pdf. [file ALL-81-2435-s001.pdf]
